# Hippocalcin Is Required for Astrocytic Differentiation through Activation of Stat3 in Hippocampal Neural Precursor Cells

**DOI:** 10.3389/fnmol.2016.00110

**Published:** 2016-10-28

**Authors:** Min-Jeong Kang, Shin-Young Park, Joong-Soo Han

**Affiliations:** ^1^Department of Biomedical Sciences, Graduate School of Biomedical Science and Engineering, Hanyang UniversitySeoul, South Korea; ^2^Department of Biochemistry and Molecular Biology, Biomedical Research Institute, College of Medicine, Hanyang UniversitySeoul, South Korea

**Keywords:** hippocalcin (Hpca), hippocampal neural precursor cells, astrocyte, differentiation, signal transducer and activation of transcription 3 (STAT3)

## Abstract

Hippocalcin (Hpca) is a neuronal calcium sensor protein expressed in the mammalian brain. However, its function in neural stem/precursor cells has not yet been studied. Here, we clarify the function of Hpca in astrocytic differentiation in hippocampal neural precursor cells (HNPCs). When we overexpressed Hpca in HNPCs in the presence or absence of bFGF, expression levels of nerve-growth factors such as neurotrophin-3 (NT-3), neurotrophin-4/5 (NT-4/5), and brain-derived neurotrophic factor (BDNF), together with the proneural basic helix loop helix (bHLH) transcription factors NeuroD and neurogenin 1 (Ngn1), increased significantly. In addition, there was an increase in the number of cells expressing glial fibrillary acidic protein (GFAP), an astrocyte marker, and in branch outgrowth, indicating astrocytic differentiation of the HNPCs. Downregulation of Hpca by transfection with Hpca siRNA reduced expression of NT-3, NT-4/5, BDNF, NeuroD, and Ngn1 as well as levels of GFAP protein. Furthermore, overexpression of Hpca increased the phosphorylation of STAT3 (Ser727), and this effect was abolished by treatment with a STAT3 inhibitor (S3I-201), suggesting that STAT3 (Ser727) activation is involved in Hpca-mediated astrocytic differentiation. As expected, treatment with Stat3 siRNA or STAT3 inhibitor caused a complete inhibition of astrogliogenesis induced by Hpca overexpression. Taken together, this is the first report to show that Hpca, acting through Stat3, has an important role in the expression of neurotrophins and proneural bHLH transcription factors, and that it is an essential regulator of astrocytic differentiation and branch outgrowth in HNPCs.

## Introduction

Neurons, astrocytes, and oligodendrocytes are generated from precursor cells in the neuroepithelium during development of the central nervous system (CNS) (McKay, 1997; Gage, 2000; Anderson, 2001; Merkle and Alvarez-Buylla, 2006). In the mouse cerebral cortex, neurogenesis commences around embryonic day (E) 11, peaks at around E15, and finishes around birth. While cortical astrocytes are first apparent at E16 and oligodendrocytes are present around birth, the vast majority of both cell types are produced during early neonatal development (McConnell, 1995; Qian et al., 2000). Although the separate timing of neurogenesis and gliogenesis in the CNS has been known for many years, several key questions remain to be addressed regarding the processes by which precursors differentiate into the distinct cell types.

Hippocalcin (Hpca) is a calcium-binding protein mostly found in pyramidal cells of the hippocampus (Saitoh et al., 1993; Palmer et al., 2005; Amici et al., 2009). It belongs to the family of EF-hand-containing neuronal calcium sensor (NCS) proteins that possess a Ca^2+^/myristoyl switch allowing translocation to membranes in response to increased cytosolic Ca^2+^ concentration (Kobayashi et al., 1993; Braunewell and Gundelfinger, 1999; O’Callaghan et al., 2002; O’Callaghan et al., 2003; Aravind et al., 2008). In neuronal cells, Hpca is localized in the cytoplasm and plasma membrane of cell bodies and dendrites (Saitoh et al., 1993). We previously reported that it is involved in the PKC-independent Ca^2+^-mediated PLD activation pathway (Hyun et al., 2000) and plays a key role in basic fibroblast growth factor (bFGF)-induced expression of NeuroD, leading to neurite outgrowth in H19-7 cells (Oh et al., 2008). However, the precise mechanism by which Hpca affects neurogenic or astrogenic potential has not been studied.

The neurotrophins comprise a family of four structurally related proteins; nerve growth factor (NGF), brain-derived neurotrophic factor (BDNF), neurotrophin-3 (NT-3), and neurotrophin-4 (NT-4) that exert their effects through high-affinity tyrosine kinase receptors (Trk receptors) (McAllister et al., 1999; Huang and Reichardt, 2001; Chao, 2003). Both the neurotrophins and their receptors are most highly expressed in the developing nervous system during active neuronal growth and differentiation (McAllister et al., 1999). In some cell lines, the relative levels of BDNF and NT-3 may reflect the reciprocal expression of these genes in the developing hippocampus or the differential expression observed for adult hippocampal regions (Ernfors et al., 1990; Maisonpierre et al., 1990). Although multiple effects of neurotrophins in the nervous system have been elucidated, their relevance to Hpca-mediated differentiation remains largely undefined.

Signal transducer and activation of transcription 3 (STAT3) is a key intermediary in signaling by many cytokines and growth factor receptors; it is activated by phosphorylation at Tyr705, thereby inducing dimerization, nuclear translocation and DNA binding (Henze et al., 1996; Mitchell and John, 2005). STAT3 activation is also regulated by phosphorylation at Ser727 via the MAPK or mTOR pathway (Park et al., 2010). STAT3 plays an important role in the differentiation of neurons in response to neurotrophic factors and growth factors (Yu et al., 2009). During brain development, activated STAT3 in conjunction with other cofactors induces expression of glial fibrillary acidic protein (GFAP) in neural precursor cells (Bonni et al., 1997; Nakashima et al., 1999; Takizawa et al., 2001; Wang et al., 2015). In addition, STAT3 signaling is up-regulated in certain neurodegenerative diseases; for example, spinal cord microglia and reactive astrocytes have increased levels of phosphorylated STAT3 (Shibata et al., 2009). Thus STAT3 activation could be critical for astrocytic differentiation in hippocampal neural precursor cells (HNPCs).

In this study, we found that Hpca expression increased during the differentiation of HNPCs and that overexpression of Hpca stimulated astrocytic differentiation. Overexpression of Hpca also increased the expression of proneural bHLH transcription factors and neurotrophins. In contrast, Hpca deficiency decreased GFAP expression, resulting in defects in astrogliogenesis with decreased expression of neurotrophins, reduced numbers of GFAP-positive astrocytes and lower branch lengths. Furthermore, we found that Hpca specifically promoted astrocytic differentiation by activating STAT3 (Ser727) in HNPCs.

## Materials and Methods

### Materials

Dulbecco’s modified Eagle medium/Ham’s nutrient mixture F-12 (DMEM/F-12 1:1 mixture), penicillin/streptomycin solution, NB (neurobasal medium), human insulin, and B27 were purchased from Gibco (Grand Island, NY, USA), and bFGF was from R&D Systems (Minneapolis, MN, USA). S3I-201 was obtained from Merck Calbiochem/EMD Biosciences (Darmstadt, Germany). Antibodies used were as follows: NT-3, NT-4/5, BDNF, Neurogenin 1, β-actin, and GAPDH were from Santa Cruz Biotechnology (Santa Cruz, CA, USA); p-STAT3 (Ser 727) and STAT3 were from Cell Signaling Technology (Beverly, MA, USA); Hpca and NeuroD were from Abcam (Cambridge, UK); Myc (Bethyl Laboratories, Montgomery, TX, USA); GFAP (Dako, Glostrup, Denmark); Alexa Fluor^®^ 488 secondary antibody goat anti-mouse IgG, Alexa Fluor^®^ 594 secondary antibody goat anti-rabbit IgG, and GFP monoclonal antibody were from Life Technologies (Paisley, UK). All other chemicals were of analytical grade.

### Primary Culture of Hippocampal Neural Precursor Cells

Pregnant Sprague–Dawley (SD) rats were obtained from Orient Bio Inc. (Seoul, Korea). Briefly, embryonic hippocampi were mechanically dissected from E16.5 rat embryos in chilled Hank’s balanced salt solution (Gibco), seeded at 2 × 10^5^ cells on 10-cm culture dishes (Nunc A/S, Roskilde, Denmark) precoated with 15 μg/ml poly-L-ornithine (Sigma-Aldrich, St Louis, Mo, USA) and 1 μg/ml fibronectin (Invitrogen, Carlsbad, CA, USA), then cultured for 4 days in serum-free N2 medium supplemented with 20 ng/ml bFGF (R&D Systems). The medium was changed every other day, while the bFGF was supplemented every day in order to expand the population of proliferative precursors. For high density cultures, the cells were plated at 4 × 10^4^ cells per well on coated 24-well plate, 6 × 10^5^ cells per well on coated 6-well or 1 × 0^6^ cells on coated 6-cm culture dishes. The plated cells were induced to additional proliferation in N2+bFGF up to 70–80% cell confluence, before induction of differentiation.

### Immunofluorescence Staining

Cells were fixed with 4% (w/v) paraformaldehyde in phosphate-buffered saline (PBS) for 20 min followed by washing with 0.1% BSA in PBS three times at room temperature. They blocked with 10% normal goat serum in 0.1% BSA in PBS containing 0.3% Triton X-100 for 1 h at room temperature. Next, the cells were immunostained with following primary antibodies; 1:400 rabbit polyclonal anti-GFAP (Dako), and 1:400 mouse monoclonal anti-GFP (Life Technologies) at 4°C. Cells were then labeled with 1:2000 Alexa Fluor^®^ 488 secondary antibody goat anti-mouse IgG (Life Technologies), and 1:2000 Alexa Fluor^®^ 594 secondary antibody goat anti-rabbit IgG (Life Technologies) for 1 h, before mounting in Vectashield (Vector Laboratories, Burlingame, CA, USA) with 4, 6-diamidino-2-phenylindole (DAPI) mounting medium. Immunoreactive cells were analyzed under an epifluorescence microscope (Nikon Instruments, Melville, NY, USA) at magnifications varying from ×200 to ×400.

### Transient Transfection of HNPCs

Transfection of HNPCs was carried with the Amaxa Nucleofector^®^ Kit V (Amaxa Biosystems, Köln, Germany) according to the manufacturer’s optimized protocol. In brief, HNPCs were dissociated using 0.05% trypsin for 5 min followed by the addition of 10% FBS in N2 medium. The resuspended cells were counted using a hemocytometer and 5 × 10^6^ cells aliquots were placed in separate tubes (1 tube per nucleofection). The cells were centrifuged at 1000 rpm for 5 min and resuspended in 100 μl of pre-warmed Amaxa Nucleofector Solution. Each 100 μl of cell suspension was mixed with either 10 μg each of pMSCV-IRES-EGFP or pMSCV-Hpca-myc-IRES-EGFP. These DNA-cell suspensions were immediately transferred to Amaxa certified cuvettes and electroporated using the Nucleofector program G-13. The samples were examined by RT-PCR and western blot [see below].

### RNA Interference

For Hpca or Stat3 silencing experiments, Hpca siRNA (ON-TARGET plus SMART pool), and negative control siRNA (ON-TARGET plus non-targeting pool) were purchased from Dharmacon (Lafayette, CO, USA). Stat3 siRNA (5′-CUGUCUUUAGGCUGAUCAU-3′) was purchased from Bioneer (Daejeon, Korea). Transient siRNA transfections were performed in 6-well plates; cells were transfected with Lipofectamine RNAiMAX (Invitrogen) and 100 nM siRNA according to the manufacturer’s protocol (Invitrogen); and cells were harvested 72 h later.

### Virus Construction and Transduction

Hippocalcin cDNA was cloned into a retrovirus vector containing IRES-EGFP (obtained from Dr. Hee Yong Chung, Department of Biomedical Science, Graduate School of Biomedical Science and Engineering, Hanyang University, Seoul, Republic of Korea). Virus particles were produced by transfecting the retrovirus packaging cell line 293gpg with the vector using Lipofectamine (Invitrogen), and supernatants containing viral particles were harvested after incubation for 48 h. For virus transduction, prepared HNPCs were incubated with a viral suspension (4 × 10^6^ particles/ml) containing polybrene (1 μg/ml; Sigma-Aldrich) for 4 h, followed by transfer to bFGF-supplemented N2 medium.

### Real-Time PCR and RT (Reverse Transcription)-PCR

cDNA was prepared from total mRNA extracted from cells with TRIzol^®^ reagent (Thermo Fisher Scientific Inc., Waltham, MA, USA); 1 μg samples of RNA was reverse-transcribed using Superscript II and random primers (Invitrogen). The resulting cDNA was amplified by PCR using the primers listed in **Table 1**. PCR products were analyzed on 1.5% agarose gels and stained with loading star (Dyne Bio, Seoul, Korea). For real-time PCR, 5 μl of RT reaction product was amplified in triplicate in a final volume of 20 μl iQ^TM^ SYBR^®^ Green supermix (Bio-Rad, CA, USA). The primer sequences for real-time PCR were the same as for RT-PCR.

**Table 1:** Primers used for RT-PCR.

Gene symbol	Primer sequences (5′→3′)	Annealing Temp (°C)	Cycles	PCR product size (bp)
Hippocalcin (Hpca)	ATGGGCAAGCAGAATAGCAAG	56	30	582
	TCAGAACTGGGAAGCGCTGCT			
Nt3	GGTCAGAATTCCAGCCGATGA	55	32	515
	GGCACACACACAGGAAGTGTC			
Nt4/5	CCCTGCGTCAGTACTTCTTCGAGAC	55	32	249
	CTGGACGTCAGGCACGGCCTGTTC			
Bdnf	GTGACAGTATTAGCGAGTGGG	56	28	213
	GGGTAGTTCGGCATTGC			
NeuroD	CTCAGTTCTCAGGACGAGGA	58	32	368
	TAGTTCTTGGCCAAGCGCAG			
Ngn1	ATGCCTGCCCCTTTGGAGAC	55	32	320
	TGCATACGGTTGCGCTCGC			
Gapdh	GGCATTGCTCTCAATGACAA	60	25	165
	AGGGCCTCTCTCTTGCTCTC			

### Western Blot Analysis

Cells were lysed in 20 mM Tris–HCl (pH 7.5) containing 150 mM NaCl, 1 mM EDTA, 1 mM EGTA, 2.5 mM sodium pyrophosphate, 1% Triton X-100, 1 mM PMSF, and 1 mM Na3VO4. Protein samples (20–30 μg) were loaded onto SDS–polyacrylamide gels and transferred to nitrocellulose membranes (Amersham Pharmacia Biotech, Amersham, UK) after electrophoresis. After blocking with 5% non-fat dried milk for 1 h, membranes were incubated with primary antibodies against Hpca anti-rabbit (1:500; Abcam), Myc anti-rabbit (1:2000; Bethyl Laboratories), NT-3 anti-rabbit (1:200; Santa Cruz Biotechnology), NT-4/5 anti-rabbit (1:200; Santa Cruz Biotechnology), BDNF anti-rabbit (1:250; Santa Cruz Biotechnology), NeuroD anti-rabbit (1:500; Abcam), Neurogenin 1 anti-goat (1:200; Santa Cruz Biotechnology), phospho-STAT3 anti-rabbit (1:1000; Cell Signaling Technology), STAT3 anti-mouse (1:1000; Cell Signaling Technology), β-actin anti-mouse (1:1000; Santa Cruz Biotechnology), GAPDH anti-rabbit (1:1000; Santa Cruz Biotechnology), GFAP anti-mouse (1:1000; Cell Signaling Technology) followed by HRP-conjugated secondary antibody (1:2000; New England Biolabs, Beverly, MA, USA), and specific bands were detected by ECL (Thermo Scientific, Rockford, IL, USA). Bands were quantified using ImageJ (v1.46r, National Institutes of Health, Bethesda, MA, USA).

### Cell Counting and Measurement of Branch Outgrowth

Cell counting was performed in microscopic field, using an eyepiece grid at a final magnification of 200× or 400×. Immunostained and DAPI-stained cells were counted in 5–10 random areas of each culture coverslip. All values were confirmed in at least five independent cultures. Photos of the cells were taken with an epifluorescence microscope (Nikon Instruments), and morphological characteristics were quantitated using Image J software (NIH^1^). The length of branches was defined as the distance from the cell body (proximal) to the tip of the longest branch (distal). For each graph, data on branch length were generated from at least five independent sets of astrocytes (in each condition) and greater than 100 cells were counted for each condition in each experiment.

### Statistical Analysis

Quantitative data are expressed as means ± SD of three to five independent experiments. The statistical significance of differences between groups was analyzed using Student’s *t*-test and was considered significant at *p* < 0.05.

## Results

### Hpca is Required for Glial Fibrillary Acidic Protein (GFAP) Expression during Differentiation of HNPCs

**FIGURE 1:**
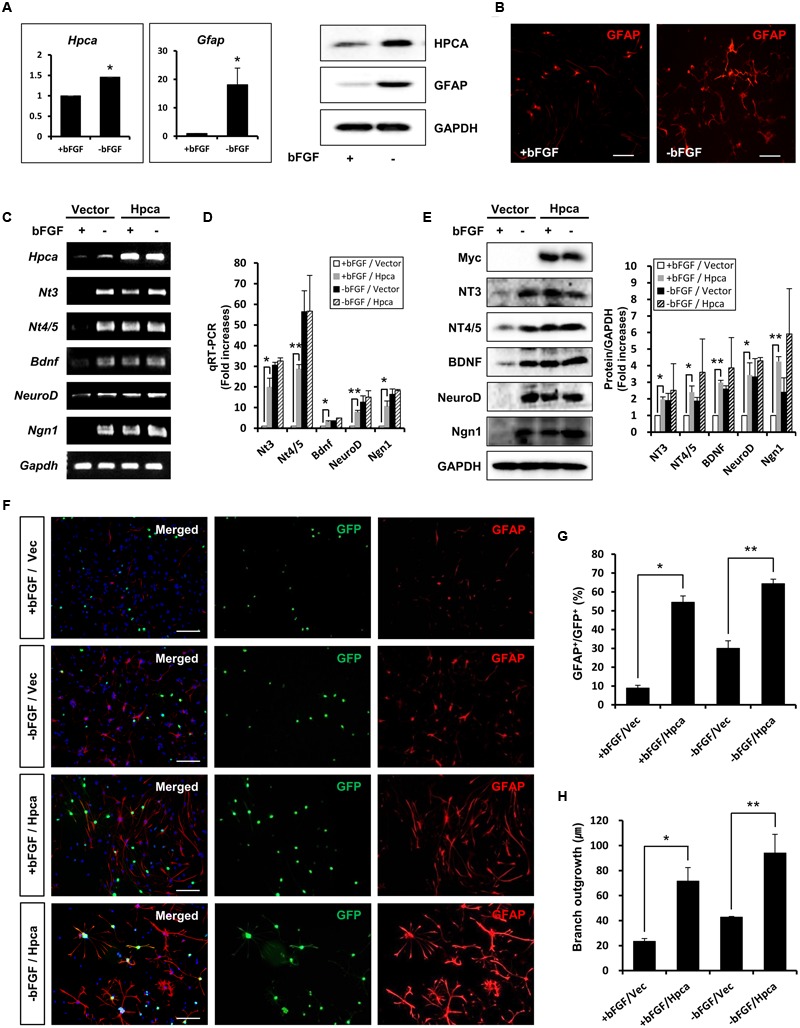
**Effect of Hippocalcin (Hpca) on GFAP expression during differentiation of HNPCs. (A,B)** HNPCs were induced to differentiate by withdrawal of bFGF. **(A)** After 48 h, mRNA levels of Hpca and Gfap were determined by real-time PCR. *^∗^p* < 0.001 compared with the +bFGF control. 20 μg aliquots of protein were analyzed by western blotting with anti-hippocalcin, anti-GFAP, and anti-GAPDH. **(B)** The cells were stained with anti-GFAP (red). Bar, 100 μm. **(C–E)** Cells were transfected with pMSCV-IRES-EGFP or pMSCV-Hpca-myc-IRES-EGFP for 48 h and then incubated for 24 h after removal of bFGF. The mRNA levels of neurotrophins and proneural bHLH transcription factors were analyzed by RT-PCR **(C)** and real-time PCR **(D)**. Data are means ± SD of three independent experiments. *^∗^p* < 0.05; *^∗∗^p* < 0.01 compared with the +bFGF/vector control. **(E)** Proteins were analyzed by western blotting with anti-Myc, anti-NT-3, anti-NT-4/5, anti-BDNF, anti-NeuroD, anti-Neurogenin 1, and anti-GAPDH. Graph shows mean densities as fold increases in three independent experiments (mean ± SD). Band intensities were quantified with Quantity Ones software. *^∗^p* < 0.05; *^∗∗^p* < 0.01 compared with the +bFGF/vector control. **(F)** HNPCs were transduced with EGFP vector or Hpca-myc-EGFP for 48 h and induced to differentiate by withdrawal of bFGF. After differentiation for 72 h, fixed cells were immunostained with anti-GFP (green) and anti-GFAP (red). GFP-stained cells fluoresce green, and GFAP-stained cells fluoresce red, while cells stained with both GFP and GFAP fluoresce yellow. Bar, 100 μm. **(G)** Graph shows the percentages of GFAP-positive cells. GFAP-positive cells among GFP-positive cells were counted under a fluorescence microscopy. The proportions of GFAP-positive cells were determined in randomly selected areas from five cultures (means ± SD). *^∗^p* < 0.001 compared with the +bFGF/vector control. *^∗∗^p* < 0.001 compared with the –bFGF/vector control. **(H)** Branch lengths were measured in random areas from five cultures (means ± SD). *^∗^p* < 0.01 compared with the +bFGF/vector control. *^∗∗^p* < 0.01 compared with the –bFGF/vector control.

Dissected and mechanically dissociated cells from hippocampi of E16.5 rat embryos were used to isolate NPCs. For growth of these cells, fresh bFGF (20 ng/ml) was present to prevent differentiation and promote proliferation. To investigate the role of Hpca in HNPCs differentiation, bFGF was removed for 48 h. We initially performed real-time PCR analyses and western blotting to determine the mRNA expression and protein level of Hpca. As shown in **Figure 1A**, Hpca and Gfap expression increased in differentiating cultures. When the cells were immunostained with GFAP, we also observed significantly increased GFAP positive cells during differentiation (**Figure 1B**). Increased levels of nerve-growth factors such as NT-3, NT-4/5, and BDNF, together with the proneural bHLH transcription factors neuroD and Ngn1 have been detected in the developing and adult hippocampus and they have major roles in development and maintenance of the hippocampal formation (Ip et al., 1993). Since these factors are closely associated with neural precursor cell differentiation, they can be used as markers of this process (Markus et al., 2002). To examine the effect of Hpca on differentiation, either pMSCV-IRES-EGFP or pMSCV-Hpca-myc-IRES-EGFP was transfected into the HNPCs for 2 days, and then cells were induced differentiation for 1 day. We showed that mRNA levels (**Figures 1C,D**) and protein expression (**Figure 1E**) of NT-3, NT-4/5, BDNF, NeuroD, and Ngn1 were significantly increased by Hpca overexpression in the presence of bFGF compared with vector control in the presence of bFGF. HNPCs have been considered as the primary progenitor cells for the neuronal and glial cell lineages during development (Rietze et al., 2001). Therefore, we examined the role of Hpca in expression of neuronal and glial markers during differentiation. HNPCs were transduced with either pMSCV-IRES-EGFP or pMSCV-Hpca-myc-IRES-EGFP for 2 days and allowed to differentiate for 3 days. We measured the percentage of GFAP-positive cells and Tuj1-positive cells under a fluorescence microscope. As shown in **Figures 1F,G**, Hpca overexpression resulted in markedly enhanced GFAP-positive astrocytes compared with vector control in the presence or absence of bFGF in HNPCs; at the same time Tuj1-positve neurons were not altered (data not shown). Moreover, Hpca overexpression significantly increased the length of branches compared with vector control under the either with bFGF or withdrawal bFGF (**Figures 1F,H**). We also found that overexpression of Hpca increased protein levels of GFAP compared with vector control in the presence or absence of bFGF in HNPCs (Supplementary Figure 1). These findings collectively imply that Hpca, which regulates the expression of neurotrophins and proneural bHLH transcription factors, promotes astrocytic differentiation of HNPCs.

### Knockdown of Hpca Suppresses the Astrocytic Differentiation of HNPCs

**FIGURE 2:**
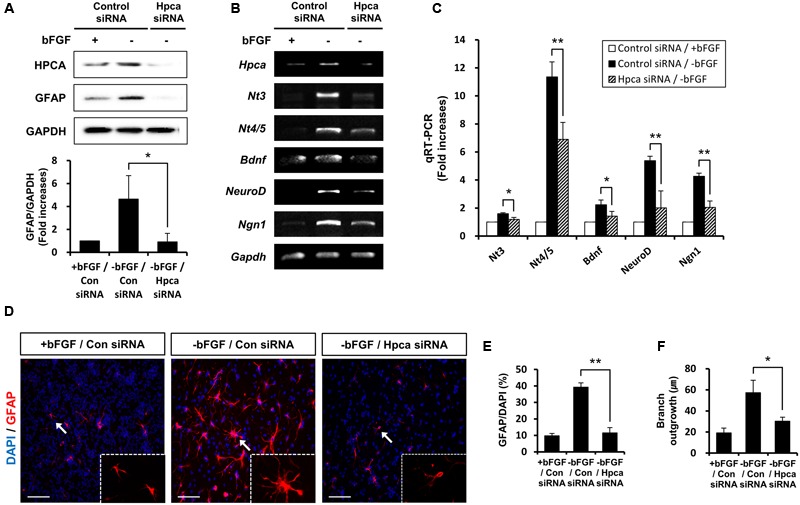
**Effect of Hpca downregulation on astrocytic differentiation of HNPCs. (A–C)** HNPCs were transfected with control siRNA or Hpca siRNA for 72 h and induced to differentiate by removal of bFGF for 24 h. **(A)** Cells were lysed and analyzed by western blotting with anti-hippocalcin, anti-GFAP, and anti-GAPDH. Graph shows mean densities as fold increases in three independent experiments (means ± SD). Band intensities were quantified with Quantity Ones^®^ software. *^∗^p* < 0.05 compared with the -bFGF/control siRNA. The mRNA levels of neurotrophins and proneural bHLH transcription factors were analyzed by RT-PCR **(B)** and real-time PCR **(C)**. Data are means ± SD of three independent experiments. *^∗^p* < 0.05; *^∗∗^p* < 0.01 compared with the -bFGF/control siRNA. **(D)** Cells were transiently transfected with control siRNA or Hpca siRNA for 72 h and induced to differentiate by withdrawal of bFGF. After differentiation for 72 h, fixed cells were stained for immunocytochemical analysis of an astrocyte marker (GFAP, red) and anti-DAPI (blue). Bar, 100 μm. The boxed area is magnified and the arrow indicates magnified cells. **(E,F)** The number of GFAP-positive cells and total cells were counted and branch lengths were measured in randomly selected areas from five cultures (means ± SD). *^∗^p* < 0.05; *^∗∗^p* < 0.001 compared with the -bFGF/control siRNA.

To confirm the role of Hpca in the expression of GFAP, we examined the effects of Hpca knockdown. 3 days after transfection of HNPCs with Hpca siRNA, the cells were allowed to differentiate for 1 day, and Hpca expression was examined by western blotting. As shown in **Figure 2A**, Hpca siRNA markedly decreased expression of GFAP. We also found that knockdown of Hpca decreased expression of NT-3, NT-4/5, BDNF, NeuroD, and Ngn1 in the absence of bFGF in HNPCs (**Figures 2B,C**). To confirm the effect of Hpca on astrocytic differentiation and branch outgrowth of HNPCs, either control siRNA or Hpca siRNA was transfected into the HNPCs for 3 days. Similarly, as shown in **Figures 2D,E**, it reduced the number of GFAP-positive cells as well as branch outgrowth (**Figures 2D,F**).

Taken together, these results indicate that Hpca is crucial for the expression of GFAP and for branch formation.

### Hpca Increases Activation of Stat3 (Ser727) in Hnpcs

**FIGURE 3:**
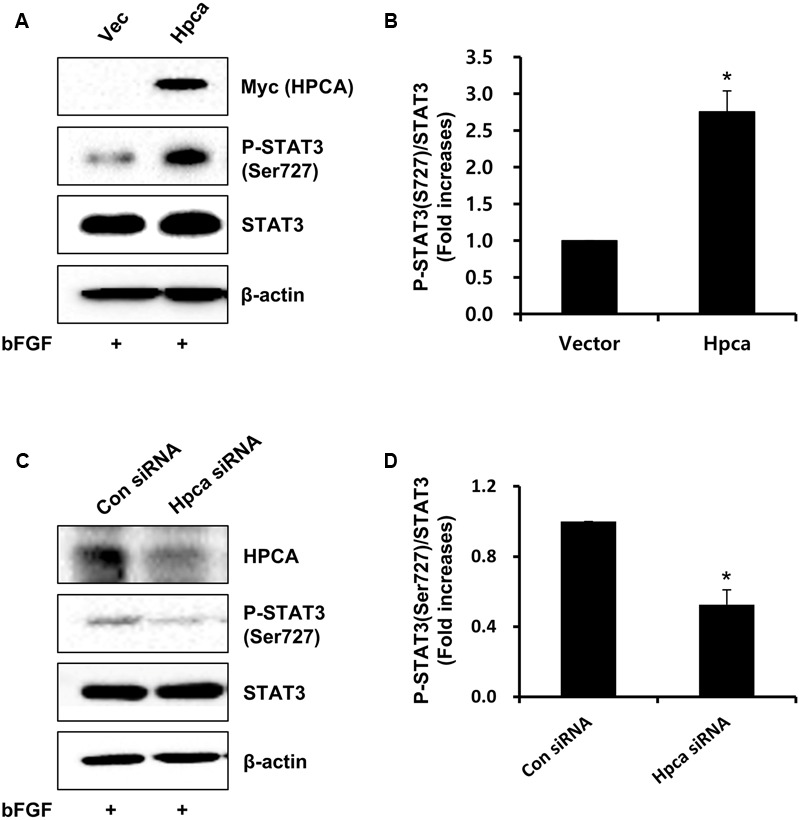
**Effect of Hpca on STAT3 (Ser727) activation in HNPCs. (A)** HNPCs were transfected with pMSCV-IRES-EGFP or pMSCV-Hpca-myc-IRES-EGFP for 48 h in the presence of bFGF. Proteins were analyzed by western blotting with anti-Myc, anti-p-STAT3 (Ser727), anti-STAT3, and anti-β-actin. **(B)** Graph shows mean densities as fold increases in three independent experiments (means ± SD). Band intensities were quantified with Quantity Ones^®^ software. *^∗^p* < 0.001 compared with the vector control. **(C)** Cells were transfected with control siRNA or Hpca siRNA for 72 h. Cell were lysed and analyzed by western blotting with anti-hippocalcin, anti-p-STAT3 (Ser727), anti-STAT3, and anti-β-actin. **(D)** Graph shows mean densities as fold increases from three independent experiments (means ± SD). *^∗^p* < 0.001 compared with the +bFGF/control siRNA.

We next asked which signaling pathway was responsible for Hpca-mediated astrocytic differentiation of HNPCs. Because the STAT3 activation is associated with GFAP expression in neural precursor cells (Sun et al., 2001), we investigated whether STAT3 was phosphorylated by Hpca overexpression. As shown in **Figures 3A,B**, phosphorylation of STAT3 at Ser727 was significantly increased by overexpression of Hpca compared to vector control in HNPCs. On the other hand, knockdown of Hpca decreased the phosphorylation of STAT3 (Ser727) (**Figures 3C,D**). These results suggest that Hpca has an important role in the activation of STAT3 (Ser727) during astrocytic differentiation of HNPCs.

### Inhibition of STAT3 (Ser727) Activation Reduces Astrocytic Differentiation of HNPCs

**FIGURE 4:**
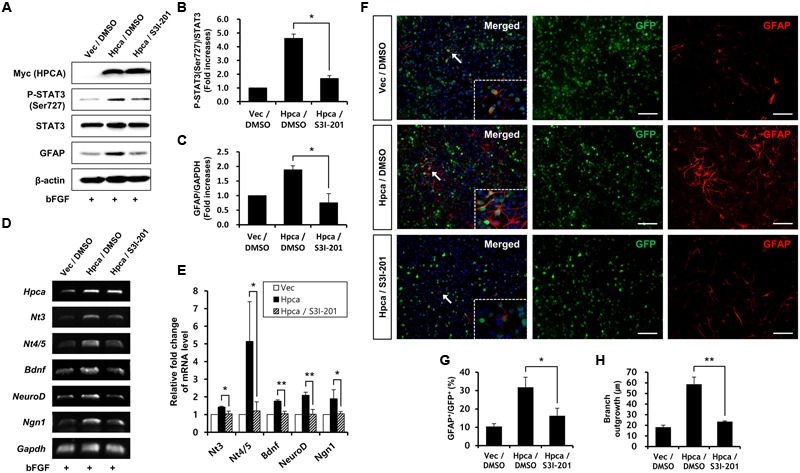
**Inhibitory effect of STAT3 (Ser727) activation on astrocytic differentiation of HNPCs. (A)** HNPCs were transfected with pMSCV-IRES-EGFP or pMSCV-Hpca-myc-IRES-EGFP for 48 h and then treated with 10 μM S3I-201 for 24 h. Cells were lysed and analyzed by western blotting with anti-Myc, anti-p-STAT3 (Ser727), anti-STAT3, anti-GFAP, and anti-β-actin. **(B,C)** Graphs show mean densities as fold increases from three independent experiments (means ± SD). Band intensities were quantified with Quantity Ones^®^ software. *^∗^p* < 0.01 compared with the Hpca/DMSO. **(D,E)** Cells were transfected with pMSCV-IRES-EGFP or pMSCV-Hpca-myc-IRES-EGFP for 48 h and then treated with 10 μM S3I-201 for 24 h. mRNA levels of neurotrophins and proneural bHLH transcription factors were measured by RT-PCR **(D)** and real-time PCR **(E)**. Graph shows mean densities as fold increases in three independent experiments (means ± SD). *^∗^p* < 0.05; *^∗∗^p* < 0.01 compared with the Hpca/DMSO. **(F)** Cells were transduced with EGFP vector or Hpca-myc-EGFP for 48 h and then treated with 10 μM S3I-201 for 24 h. Fixed cells were immunostained with anti-GFP (green) and anti-GFAP (red). Bar, 100 μm. The boxed area is magnified and the arrow indicates magnified cells. **(G,H)** GFAP-positive cells were counted under a fluorescence microscopy and branch lengths were measured in randomly selected areas from five cultures. Data are means ± SD of five values. *^∗^p* < 0.05; *^∗∗^p* < 0.001 compared with the Hpca/DMSO.

**FIGURE 5:**
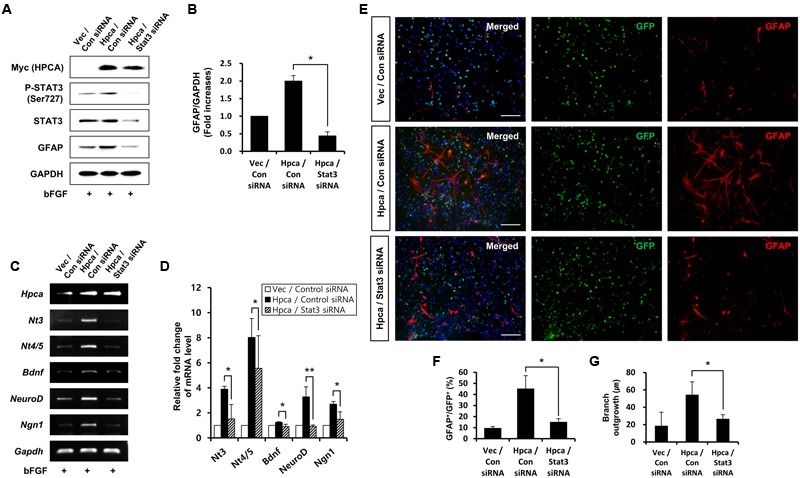
**Effect of STAT3 downregulation on astrocytic differentiation of HNPCs. (A)** HNPCs were transfected with pMSCV-IRES-EGFP or pMSCV-Hpca-myc-IRES-EGFP for 48 h and then transfected with control siRNA or Stat3 siRNA for 72 h. Proteins were analyzed in western blotting with anti-Myc, anti-p-STAT3 (Ser727), anti-STAT3, anti-GFAP, and anti-GAPDH. **(B)** Graph shows mean densities as fold increases from three independent experiments (means ± SD). *^∗^p* < 0.01 compared with Hpca/control siRNA. **(C,D)** mRNA levels of neurotrophins and proneural bHLH transcription factors were analyzed by RT-PCR **(C)** and real-time PCR **(D)**. Data are means ± SD of three independent experiments. *^∗^p* < 0.05; *^∗∗^p* < 0.01 compared with Hpca/control siRNA. **(E)** Cells were transduced with EGFP vector or Hpca-myc-EGFP for 48 h and then transfected with control siRNA or Stat3 siRNA for 72 h. Fixed cells were immunostained with anti-GFP (green) and anti-GFAP (red). Bar, 100 μm. **(F)** Graph shows the percentages of GFAP-positive cells. GFAP-positive cells among GFP-positive cells were counted under fluorescence microscopy. The proportions of GFAP-positive cells were determined in randomly selected areas from five cultures (means ± SD). *^∗^p* < 0.05 compared with the Hpca/control siRNA. **(G)** Branch lengths were measured in randomly selected areas from five cultures (means ± SD). *^∗^p* < 0.05 compared with the Hpca/control siRNA.

Next, we determined whether STAT3 (Ser727) activation is required for astrocytic differentiation of HNPCs. Cells were transiently transfected with pMSCV-IRES-EGFP or pMSCV-Hpca-myc-IRES-EGFP for 2 days and treated with the STAT3 inhibitor, S3I-201, for 1 day. Inhibition of STAT3 not only decreased phosphorylation of STAT3 (Ser727) (**Figures 4A,B**) but also reduced the level of GFAP (**Figures 4A,C**) and transcript levels of NT-3, NT-4/5, BDNF, NeuroD, and Ngn1 (**Figures 4D,E**). We also found that treatment with 10 μM S3I-201 significantly reduced the number of GFAP-positive cells (31.8 ± 1.8% vs. 16.3 ± 1.3%, *p* < 0.05; **Figures 4F,G**) compared to the overexpression of Hpca. To test if STAT3 can also promote the branch outgrowth, we calculated the length of branches. The extent of branch outgrowth observed upon overexpression of Hpca was also reduced when STAT3 was inhibited (**Figures 4F,H**). To further determine the involvement of STAT3 in Hpca-mediated astrocytic differentiation, we knocked down STAT3 using siRNA. Stat3 siRNA completely abolished expression of GFAP (**Figures 5A,B**) and transcripts of NT-3, NT-4/5, BDNF, NeuroD, and Ngn1 compared with overexpression of Hpca (**Figures 5C,D**). We also found that knockdown of STAT3 markedly decreased the number of GFAP-positive cells (45.0 ± 4.0% vs. 15.0 ± 1.0%, *p* < 0.05; **Figures 5E,F**) compared with overexpression of Hpca. Furthermore, knockdown of STAT3 decreased branch outgrowth (**Figure 5G**). Taken together, our results demonstrate that STAT3 is the signaling molecule that triggers branch outgrowth leading to astrocytic differentiation of HNPCs.

## Discussion

The objective of this study was to elucidate the role of Hpca in neural precursor cell differentiation during hippocampal development. Differentiation of HNPCs was accompanied by upregulation of Hpca expression, and experiments involving gain or loss of Hpca function argued that Hpca was important for the differentiation process. Development of the mammalian nervous system is fulfilled through precursor cells that sequentially generate neurons and glial cells (Luskin et al., 1988; Price and Thurlow, 1988; Walsh and Cepko, 1988; Davis and Temple, 1994). *In vivo*, cortical neurogenesis occurs during embryogenesis, while gliogenesis largely occurs postnatally. Remarkably, this precisely timed process can be reproduced in isolated embryonic cortical stem cells (Qian et al., 2000; Ross et al., 2003; Miller and Gauthier, 2007). Therefore, a major question is how the fates of neural precursors are switched from neurogenesis in the earlier stages to gliogenesis later. One possible explanation is that the precursor cells themselves could change, for example, in response to neurogenic factors (Nieto et al., 2001; Sun et al., 2001; Menard et al., 2002; He et al., 2005), or growth factors (Sauvageot and Stiles, 2002). Here, we found that Hpca controlled the competence of precursors to differentiate into astrocytes by regulating the expression of proneural bHLH transcription factors and neurotrophins that induce GFAP expression in HNPCs. To our knowledge, this is the first example in which Hpca promoted differentiation toward a specific lineage, and particularly, toward astrocytes during development.

Hippocalcin has an important role in AMPAR-mediated endocytosis and NMDAR-mediated hippocampal LTD (Palmer et al., 2005), and its expression is involved in synapse formation and age-related postsynaptic function (Furuta et al., 1999). We have reported that it is one of the major regulatory proteins in the Ca^2+^-mediated PLD signaling pathway (Oh et al., 2006) and that it induces neurite outgrowth leading to neuronal differentiation in H19-7 cells (Oh et al., 2008). However, its involvement in astrocytic differentiation has not been studied. In this study, we found that expression of neurotrophins and proneural bHLH transcription factors are regulated by Hpca-mediated activation of STAT3 (Ser727), which leads to branch outgrowth and astrocytic differentiation in HNPCs. Several studies have reported that expression of neurotrophins was increased in cultured rat hippocampal astrocytes and astrocytes showed expression of neurotrophin receptors. However, the ability of neurotrophins to induce astrocytic differentiation and the effect of proneural bHLH transcription factors on astrocytic differentiation has not been studied. Thus, we will investigate whether Hpca-mediated expression of neurotrophins and proneural bHLH transcription factors has effect on astrocytic differentiation of HNPCs in further study.

The determination of neuronal/glial fate during CNS development involves complex interactions between intrinsic signals acting through many transcription factors (Edlund and Jessell, 1999). Above all, STAT3 plays essential roles in determining the fate of neural stem cells (NSCs) (Han et al., 2006). Inhibition of STAT3 enhances the differentiation of human NSCs to motor neurons and reduces astrocytosis, and similarly, induces neurogenesis and inhibits gliogenesis (Gu et al., 2005; Cao et al., 2010; Natarajan et al., 2014). Previous studies have shown that GFAP expression in neural precursor cells is dependent on the activation of STAT3 (Bonni et al., 1997; Nakashima et al., 1999; Takizawa et al., 2001; Herrmann et al., 2008). Interestingly, we found that Hpca enhanced STAT3 (Ser727) activation, but did not cause any phosphorylation at Tyr705 (data not shown). Furthermore, inhibition of STAT3 activation by Stat3 siRNA or STAT3 inhibitor decreased transcripts of neurotrophins and proneural bHLH transcription factors and GFAP expression induced by Hpca overexpression, indicating that Hpca-mediated STAT3 (Ser727) activation is essential for astrocytic differentiation. Our results are consistent with earlier reports in which suppression or conditional deletion of STAT3 inhibited glial differentiation (Sun et al., 2001; Gu et al., 2005; Natarajan et al., 2014). However, the kinase involved in Hpca-mediated STAT3 activation remains to be identified. Interestingly, the STAT3 inhibitor and Stat3 siRNA in our experiments completely inhibit the expression of neurotrophins and proneural bHLH transcription factors. Recent studies have shown that the activities of STAT family proteins can be modulated by interactions with other DNA-binding sites; for instance, STAT3 interacts with c-Jun, c-Fos and the CREB-binding protein co-activators (Zhang et al., 1999; Coqueret and Gascan, 2000). Although the mechanisms underlying this process have not been fully elucidated, our findings offer a theoretical basis for how astrocytic differentiation of HNPCs is regulated.

Taken together, the present study provides the first evidence that Hpca promotes gliogenesis in HNPCs, and induces GFAP expression and branch outgrowth by activating STAT3 (Ser727). Gliogenesis is tightly controlled because of its critical importance in proper physiological function, and multiple signal pathways control the growth and directionality of the relevant cell-fate decision. Recent work has demonstrated abnormalities in the signaling pathways responsible for gliogenesis and neurogenesis could contribute to the pathogenesis of neurodegenerative diseases and tumor development within the nervous system (Lee et al., 2000; Shors, 2004). Recognition of the regulatory mechanisms of gliogenesis provides new direction for intervention of neurogenic disorders. Furthermore, Hpca is implicated in memory formation, neuronal excitability (Kobayashi et al., 2012) and Hpca knockout mice have a defect in spatial and associative memory (Kobayashi et al., 2005). It is possible that Hpca plays an important role in neural plasticity and memory function and further studies should address its possible use as a marker of neurodegenerative disorder and for promoting glial cell generation *in vivo*.

## Author Contributions

M-JK participated in the conception, execution and analysis of experiments and in the writing of the manuscript, then edited in the additions and changes made by the other co-authors. S-YP participated in the conception, analysis of initial experiments and revision of the manuscript. J-SH contributed to the development of the hypothesis and additional text and revision of the manuscript. All authors agree to be accountable for all aspects of the work.

## Conflict of Interest Statement

The authors declare that the research was conducted in the absence of any commercial or financial relationships that could be construed as a potential conflict of interest.

## Footnotes

1 http://rsb.Info.nih.gov/ij/

## References

[B1] Amici M. Doherty A. Jo J. Jane D. Cho K. Collingridge G. (2009). Neuronal calcium sensors and synaptic plasticity. *Biochem. Soc. Trans.* 37(Pt 6) 1359–1363. 10.1042/BST0371359 19909276

[B2] Anderson D. J. (2001). Stem cells and pattern formation in the nervous system: the possible versus the actual. *Neuron* 30 19–35. 10.1016/S0896-6273(01)00260-4 11343642

[B3] Aravind P. Chandra K. Reddy P. P. Jeromin A. Chary K. V. Sharma Y. (2008). Regulatory and structural EF-hand motifs of neuronal calcium sensor-1: Mg 2+ modulates Ca 2+ binding, Ca 2+ -induced conformational changes, and equilibrium unfolding transitions. *J. Mol. Biol.* 376 1100–1115. 10.1016/j.jmb.2007.12.033 18199453

[B4] Bonni A. Sun Y. Nadal-Vicens M. Bhatt A. Frank D. A. Rozovsky I. (1997). Regulation of gliogenesis in the central nervous system by the JAK-STAT signaling pathway. *Science* 278 477–483. 10.1126/science.278.5337.477 9334309

[B5] Braunewell K. H. Gundelfinger E. D. (1999). Intracellular neuronal calcium sensor proteins: a family of EF-hand calcium-binding proteins in search of a function. *Cell Tissue Res.* 295 1–12. 10.1007/s004410051207 9931348

[B6] Cao F. Hata R. Zhu P. Nakashiro K. Sakanaka M. (2010). Conditional deletion of Stat3 promotes neurogenesis and inhibits astrogliogenesis in neural stem cells. *Biochem. Biophys. Res. Commun.* 394 843–847. 10.1016/j.bbrc.2010.03.092 20303333

[B7] Chao M. V. (2003). Neurotrophins and their receptors: a convergence point for many signalling pathways. *Nat. Rev. Neurosci.* 4 299–309. 10.1038/nrn1078 12671646

[B8] Coqueret O. Gascan H. (2000). Functional interaction of STAT3 transcription factor with the cell cycle inhibitor p21WAF1/CIP1/SDI1. *J. Biol. Chem.* 275 18794–18800. 10.1074/jbc.M001601200 10764767

[B9] Davis A. A. Temple S. (1994). A self-renewing multipotential stem cell in embryonic rat cerebral cortex. *Nature* 372 263–266. 10.1038/372263a0 7969470

[B10] Edlund T. Jessell T. M. (1999). Progression from extrinsic to intrinsic signaling in cell fate specification: a view from the nervous system. *Cell* 96 211–224. 10.1016/S0092-8674(00)80561-9 9988216

[B11] Ernfors P. Wetmore C. Olson L. Persson H. (1990). Identification of cells in rat brain and peripheral tissues expressing mRNA for members of the nerve growth factor family. *Neuron* 5 511–526. 10.1016/0896-6273(90)90090-3 2206535

[B12] Furuta Y. Kobayashi M. Masaki T. Takamatsu K. (1999). Age-related changes in expression of hippocalcin and NVP2 in rat brain. *Neurochem. Res.* 24 651–658. 10.1023/A:1021000425070 10344594

[B13] Gage F. H. (2000). Mammalian neural stem cells. *Science* 287 1433–1438. 10.1126/science.287.5457.1433 10688783

[B14] Gu F. Hata R. Ma Y. J. Tanaka J. Mitsuda N. Kumon Y. (2005). Suppression of Stat3 promotes neurogenesis in cultured neural stem cells. *J. Neurosci. Res.* 81 163–171. 10.1002/jnr.20561 15948155

[B15] Han Y. Amin H. M. Franko B. Frantz C. Shi X. Lai R. (2006). Loss of SHP1 enhances JAK3/STAT3 signaling and decreases proteosome degradation of JAK3 and NPM-ALK in ALK+ anaplastic large-cell lymphoma. *Blood* 108 2796–2803. 10.1182/blood-2006-04-017434 16825495

[B16] He F. Ge W. Martinowich K. Becker-Catania S. Coskun V. Zhu W. (2005). A positive autoregulatory loop of Jak-STAT signaling controls the onset of astrogliogenesis. *Nat. Neurosci.* 8 616–625. 10.1038/nn1440 15852015 PMC4222251

[B17] Henze D. A. Cameron W. E. Barrionuevo G. (1996). Dendritic morphology and its effects on the amplitude and rise-time of synaptic signals in hippocampal CA3 pyramidal cells. *J. Comp. Neurol.* 369 331–344. 8743416 10.1002/(SICI)1096-9861(19960603)369:3<331::AID-CNE1>3.0.CO;2-6

[B18] Herrmann J. E. Imura T. Song B. Qi J. Ao Y. Nguyen T. K. (2008). STAT3 is a critical regulator of astrogliosis and scar formation after spinal cord injury. *J. Neurosci.* 28 7231–7243. 10.1523/JNEUROSCI.1709-08.2008 18614693 PMC2583788

[B19] Huang E. J. Reichardt L. F. (2001). Neurotrophins: roles in neuronal development and function. *Annu. Rev. Neurosci.* 24 677–736. 10.1146/annurev.neuro.24.1.677 11520916 PMC2758233

[B20] Hyun J. K. Yon C. Kim Y. S. Noh D. Y. Lee K. H. Han J. S. (2000). Role of hippocalcin in Ca2+ -induced activation of phospholipase D. *Mol. Cells* 10 669–677. 10.1007/s10059-000-0669-1 11211872

[B21] Ip N. Y. Li Y. Yancopoulos G. D. Lindsay R. M. (1993). Cultured hippocampal neurons show responses to BDNF, NT-3, and NT-4 but not NGF. *J. Neurosci.* 13 3394–3405. 7688038 10.1523/JNEUROSCI.13-08-03394.1993 PMC6576536

[B22] Kobayashi M. Hamanoue M. Masaki T. Furuta Y. Takamatsu K. (2012). Hippocalcin mediates calcium-dependent translocation of brain-type creatine kinase (BB-CK) in hippocampal neurons. *Biochem. Biophys. Res. Commun.* 429 142–147. 10.1016/j.bbrc.2012.10.125 23142228

[B23] Kobayashi M. Masaki T. Hori K. Masuo Y. Miyamoto M. Tsubokawa H. (2005). Hippocalcin-deficient mice display a defect in cAMP response element-binding protein activation associated with impaired spatial and associative memory. *Neuroscience* 133 471–484. 10.1016/j.neuroscience.2005.02.034 15878804

[B24] Kobayashi M. Takamatsu K. Saitoh S. Noguchi T. (1993). Myristoylation of hippocalcin is linked to its calcium-dependent membrane association properties. *J. Biol. Chem.* 268 18898–18904. 8360179

[B25] Lee J. C. Mayer-Proschel M. Rao M. S. (2000). Gliogenesis in the central nervous system. *Glia* 30 105–121. 10.1002/(SICI)1098-1136(200004)30:2<105::AID-GLIA1>3.0.CO;2-H 10719353

[B26] Luskin M. B. Pearlman A. L. Sanes J. R. (1988). Cell lineage in the cerebral cortex of the mouse studied in vivo and in vitro with a recombinant retrovirus. *Neuron* 1 635–647. 10.1016/0896-6273(88)90163-8 3272182

[B27] Maisonpierre P. C. Belluscio L. Friedman B. Alderson R. F. Wiegand S. J. Furth M. E. (1990). NT-3, BDNF, and NGF in the developing rat nervous system: parallel as well as reciprocal patterns of expression. *Neuron* 5 501–509. 1688327 10.1016/0896-6273(90)90089-x

[B28] Markus A. Patel T. D. Snider W. D. (2002). Neurotrophic factors and axonal growth. *Curr. Opin. Neurobiol.* 12 523–531. 10.1016/S0959-4388(02)00372-0 12367631

[B29] McAllister A. K. Katz L. C. Lo D. C. (1999). Neurotrophins and synaptic plasticity. *Annu. Rev. Neurosci.* 22 295–318. 10.1146/annurev.neuro.22.1.295 10202541

[B30] McConnell S. K. (1995). Constructing the cerebral cortex: neurogenesis and fate determination. *Neuron* 15 761–768. 10.1016/0896-6273(95)90168-X 7576626

[B31] McKay R. (1997). Stem cells in the central nervous system. *Science* 276 66–71. 10.1126/science.276.5309.66 9082987

[B32] Menard C. Hein P. Paquin A. Savelson A. Yang X. M. Lederfein D. (2002). An essential role for a MEK-C/EBP pathway during growth factor-regulated cortical neurogenesis. *Neuron* 36 597–610. 10.1016/S0896-6273(02)01026-7 12441050

[B33] Merkle F. T. Alvarez-Buylla A. (2006). Neural stem cells in mammalian development. *Curr. Opin. Cell Biol.* 18 704–709. 10.1016/j.ceb.2006.09.008 17046226

[B34] Miller F. D. Gauthier A. S. (2007). Timing is everything: making neurons versus glia in the developing cortex. *Neuron* 54 357–369. 10.1016/j.neuron.2007.04.019 17481390

[B35] Mitchell T. J. John S. (2005). Signal transducer and activator of transcription (STAT) signalling and T-cell lymphomas. *Immunology* 114 301–312. 10.1111/j.1365-2567.2005.02091.x 15720432 PMC1782085

[B36] Nakashima K. Wiese S. Yanagisawa M. Arakawa H. Kimura N. Hisatsune T. (1999). Developmental requirement of gp130 signaling in neuronal survival and astrocyte differentiation. *J. Neurosci.* 19 5429–5434. 10377352 10.1523/JNEUROSCI.19-13-05429.1999 PMC6782325

[B37] Natarajan R. Singal V. Benes R. Gao J. Chan H. Chen H. (2014). STAT3 modulation to enhance motor neuron differentiation in human neural stem cells. *PLoS ONE* 9:e100405 10.1371/journal.pone.0100405 PMC4063761 24945434

[B38] Nieto M. Schuurmans C. Britz O. Guillemot F. (2001). Neural bHLH genes control the neuronal versus glial fate decision in cortical progenitors. *Neuron* 29 401–413. 10.1016/S0896-6273(01)00214-8 11239431

[B39] O’Callaghan D. W. Ivings L. Weiss J. L. Ashby M. C. Tepikin A. V. Burgoyne R. D. (2002). Differential use of myristoyl groups on neuronal calcium sensor proteins as a determinant of spatio-temporal aspects of Ca2+ signal transduction. *J. Biol. Chem.* 277 14227–14237. 10.1074/jbc.M111750200 11836243

[B40] O’Callaghan D. W. Tepikin A. V. Burgoyne R. D. (2003). Dynamics and calcium sensitivity of the Ca2+/myristoyl switch protein hippocalcin in living cells. *J. Cell Biol.* 163 715–721. 10.1083/jcb.200306042 14638856 PMC2173692

[B41] Oh D. Y. Cho J. H. Park S. Y. Kim Y. S. Yoon Y. J. Yoon S. H. (2008). A novel role of hippocalcin in bFGF-induced neurite outgrowth of H19-7 cells. *J. Neurosci. Res.* 86 1557–1565. 10.1002/jnr.21602 18183620

[B42] Oh D. Y. Yon C. Oh K. J. Lee K. S. Han J. S. (2006). Hippocalcin increases phospholipase D2 expression through extracellular signal-regulated kinase activation and lysophosphatidic acid potentiates the hippocalcin-induced phospholipase D2 expression. *J. Cell. Biochem.* 97 1052–1065. 10.1002/jcb.20665 16294323

[B43] Palmer C. L. Lim W. Hastie P. G. Toward M. Korolchuk V. I. Burbidge S. A. (2005). Hippocalcin functions as a calcium sensor in hippocampal LTD. *Neuron* 47 487–494. 10.1016/j.neuron.2005.06.014 16102532 PMC1563146

[B44] Park S. Y. Cho J. H. Ma W. Choi H. J. Han J. S. (2010). Phospholipase D2 acts as an important regulator in LPS-induced nitric oxide synthesis in Raw 264.7 cells. *Cell. Signal.* 22 619–628. 10.1016/j.cellsig.2009.11.016 19963059

[B45] Price J. Thurlow L. (1988). Cell lineage in the rat cerebral cortex: a study using retroviral-mediated gene transfer. *Development* 104 473–482. 3151483 10.1242/dev.104.3.473

[B46] Qian X. Shen Q. Goderie S. K. He W. Capela A. Davis A. A. (2000). Timing of CNS cell generation: a programmed sequence of neuron and glial cell production from isolated murine cortical stem cells. *Neuron* 28 69–80. 10.1016/S0896-6273(00)00086-6 11086984

[B47] Rietze R. L. Valcanis H. Brooker G. F. Thomas T. Voss A. K. Bartlett P. F. (2001). Purification of a pluripotent neural stem cell from the adult mouse brain. *Nature* 412 736–739. 10.1038/35089085 11507641

[B48] Ross S. E. Greenberg M. E. Stiles C. D. (2003). Basic helix-loop-helix factors in cortical development. *Neuron* 39 13–25. 10.1016/S0896-6273(03)00365-9 12848929

[B49] Saitoh S. Takamatsu K. Kobayashi M. Noguchi T. (1993). Distribution of hippocalcin mRNA and immunoreactivity in rat brain. *Neurosci. Lett.* 157 107–110. 10.1016/0304-3940(93)90654-4 8233019

[B50] Sauvageot C. M. Stiles C. D. (2002). Molecular mechanisms controlling cortical gliogenesis. *Curr. Opin. Neurobiol* 12 244–249. 10.1016/S0959-4388(02)00322-7 12049929

[B51] Shibata N. Kakita A. Takahashi H. Ihara Y. Nobukuni K. Fujimura H. (2009). Activation of signal transducer and activator of transcription-3 in the spinal cord of sporadic amyotrophic lateral sclerosis patients. *Neurodegener. Dis.* 6 118–126. 10.1159/000213762 19372705

[B52] Shors T. J. (2004). Memory traces of trace memories: neurogenesis, synaptogenesis and awareness. *Trends Neurosci.* 27 250–256. 10.1016/j.tins.2004.03.007 15111006 PMC3363970

[B53] Sun Y. Nadal-Vicens M. Misono S. Lin M. Z. Zubiaga A. Hua X. (2001). Neurogenin promotes neurogenesis and inhibits glial differentiation by independent mechanisms. *Cell* 104 365–376. 10.1016/S0092-8674(01)00224-0 11239394

[B54] Takizawa T. Yanagisawa M. Ochiai W. Yasukawa K. Ishiguro T. Nakashima K. (2001). Directly linked soluble IL-6 receptor-IL-6 fusion protein induces astrocyte differentiation from neuroepithelial cells via activation of STAT3. *Cytokine* 13 272–279. 10.1006/cyto.2000.0831 11243705

[B55] Walsh C. Cepko C. L. (1988). Clonally related cortical cells show several migration patterns. *Science* 241 1342–1345. 10.1126/science.3137660 3137660

[B56] Wang T. Yuan W. Liu Y. Zhang Y. Wang Z. Zhou X. (2015). The role of the JAK-STAT pathway in neural stem cells, neural progenitor cells and reactive astrocytes after spinal cord injury. *Biomed. Rep.* 3 141–146. 10.3892/br.2014.401 25798237 PMC4360852

[B57] Yu Y. Ren W. Ren B. (2009). Expression of signal transducers and activator of transcription 3 (STAT3) determines differentiation of olfactory bulb cells. *Mol. Cell. Biochem.* 320 101–108. 10.1007/s11010-008-9911-5 18777086

[B58] Zhang X. Wrzeszczynska M. H. Horvath C. M. Darnell J. E. Jr (1999). Interacting regions in Stat3 and c-Jun that participate in cooperative transcriptional activation. *Mol. Cell. Biol.* 19 7138–7146. 10.1128/MCB.19.10.7138 10490649 PMC84707

