# Supplementary material for: Hippocalcin Is Required for Astrocytic Differentiation through Activation of Stat3 in Hippocampal Neural Precursor Cells
Source: Front Mol Neurosci. 2016 Oct 28;9:110. doi: 10.3389/fnmol.2016.00110 (PMC5083843; doi:10.3389/fnmol.2016.00110)
Supplement: Supplementary file 1 [file Presentation_1.PPTX]

## Slide 1
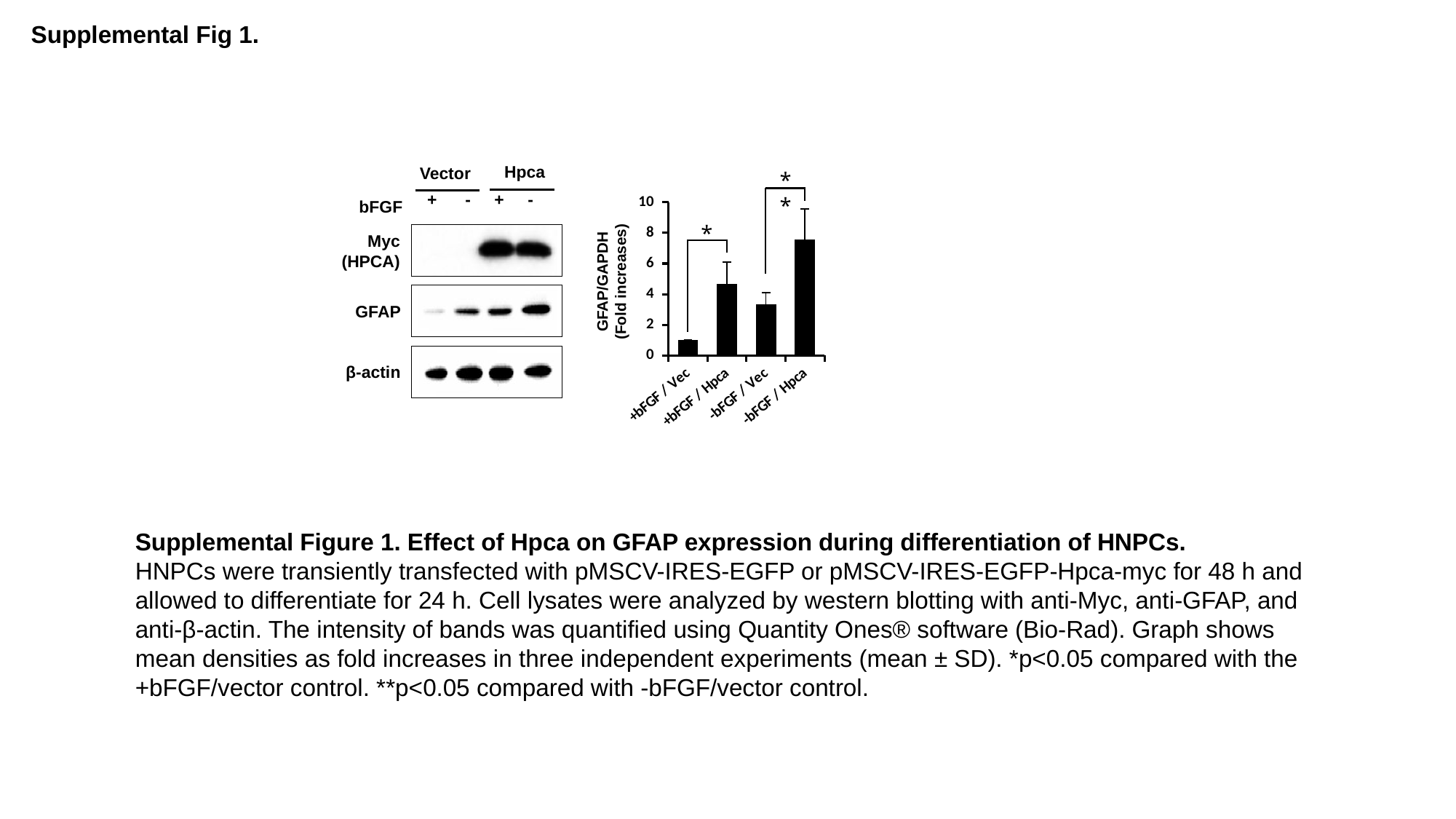

Supplemental Fig 1.
Hpca
Vector
**
### Chart
| Category | |
|---|---|
| +bFGF / Vec | 1.0 |
| +bFGF / Hpca | 4.663514574695957 |
| -bFGF / Vec | 3.327075328477131 |
| -bFGF / Hpca | 7.526558885408076 |bFGF
 + - + -
*
Myc (HPCA)
GFAP/GAPDH
(Fold increases)
GFAP
β-actin
Supplemental Figure 1. Effect of Hpca on GFAP expression during differentiation of HNPCs.
HNPCs were transiently transfected with pMSCV-IRES-EGFP or pMSCV-IRES-EGFP-Hpca-myc for 48 h and allowed to differentiate for 24 h. Cell lysates were analyzed by western blotting with anti-Myc, anti-GFAP, and anti-β-actin. The intensity of bands was quantified using Quantity Ones® software (Bio-Rad). Graph shows mean densities as fold increases in three independent experiments (mean ± SD). *p<0.05 compared with the +bFGF/vector control. **p<0.05 compared with -bFGF/vector control.
